# Microneedle Patch for In Situ Neutrophil Monitoring

**DOI:** 10.1002/advs.202417801

**Published:** 2025-05-08

**Authors:** Ziyi Lu, Ruisi Cai, Tianao Xie, Sheng Zhao, Sarun Juengpanich, Win Topatana, Shijie Li, Jiasheng Cao, Jiahao Hu, Tianen Chen, Jiachen Chen, Jicheng Yu, Tao Sheng, Wentao Zhang, Hao Wang, Jiahuan You, Yukai Shan, Yuchao Sun, Ruijing Shen, Zhengjie Zhao, Kangfan Ji, Ziqi Gao, Xinmin Yu, Xiujun Cai, Zhen Gu, Yuqi Zhang, Mingyu Chen

**Affiliations:** ^1^ Department of General Surgery, Sir Run Run Shaw Hospital Zhejiang University Hangzhou 310016 China; ^2^ State Key Laboratory of Advanced Drug Delivery and Release Systems, Zhejiang Provincial Key Laboratory for Advanced Drug Delivery Systems, College of Pharmaceutical Sciences Zhejiang University Hangzhou 310058 China; ^3^ National Engineering Research Center of Innovation and Application of Minimally Invasive Instruments Zhejiang University Hangzhou 310000 China; ^4^ Jinhua Institute of Zhejiang University Jinhua 321299 China; ^5^ MOE Key Laboratory of Macromolecular Synthesis and Functionalization, Department of Polymer Science and Engineering Zhejiang University Hangzhou 310027 China; ^6^ Institute of Fundamental and Transdisciplinary Research Zhejiang University Hangzhou 310058 China; ^7^ Department of Burns and Wound Care Center, Second Affiliated Hospital, School of Medicine Zhejiang University Hangzhou 310009 China

**Keywords:** cancer, microneedle, neutrophil monitoring, non‐invasive detection, portable device

## Abstract

Monitoring neutrophil levels plays an essential role in diagnosing infections and various diseases, as well as for managing specific treatments such as chemotherapy and radiotherapy. However, the traditional complete blood count technique is invasive and challenging for quick and frequent monitoring of neutrophil. Herein, a button‐like microneedle patch for rapid and convenient detection of neutrophil levels is reported without vascular invasion. This patch consists of a hollow microneedle array with a vacuum chamber for extraction of interstitial fluid, a hydrogel sheet for colorimetric detection of neutrophil level based on neutrophil‐specific enzyme (myeloperoxidase), and a mobile phone application for data recording and quantification. The detection ability is validated both in vitro and in vivo in a chemotherapy‐induced neutropenic rat model. Moreover, this button patch achieves precise measurement of the neutrophil levels in patients’ urine and abdominal drainage fluid. This minimally invasive colorimetric patch can potentiate in situ neutrophil monitoring without blood sampling procedures or in‐lab apparatus, which can serve as a substitution for routine blood counts especially for patients in less developed areas.

## Graphical abstract

An in situ chromogenic microneedle patch that achieves daily blood neutrophil monitoring without vascular invasion or laboratory apparatus is developed. The detection ability is well demonstrated in a chemotherapy‐induced neutropenic rat model and can be read out using a mobile phone application. Moreover, this patch can achieve precise neutrophil measurement of several clinical biofluids such as urine and abdominal drainage fluid.

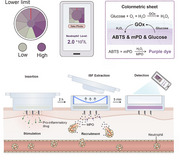

## 1 Introduction

Neutrophils act as one of the dominant responders during infection and inflammation, with the level closely correlated with the occurrence, development, and prognosis of diseases such as bacterial infections, autoimmune disorders, and certain types of leukemia.^[^ ^1^, ^2^ ^]^ Moreover, neutrophilia is usually used to distinguish bacterial infectious fever from unknown fever and neutropenia that primarily result from cancer treatments or autoimmune diseases.^[^ ^3^ ^]^ Over 20% of cancer patients who receive cytotoxic chemotherapy have suffered from neutropenia, which can lead to impaired immune function and a higher risk of serious infections.^[^ ^4^, ^5^, ^6^ ^]^ Once this happens, it is necessary to suspend chemotherapy or reduce the dose until neutrophil levels return to an acceptable range.^[^ ^7^, ^8^ ^]^ Meantime, chemotherapy should be resumed as soon as possible to minimize the interference on disease control.^[^ ^9^, ^10^ ^]^ Therefore, neutrophil levels should be strictly monitored during the entire course of chemotherapy.

Although neutrophil monitoring is widely used in the management of disease, it is primarily determined by the complete blood count (CBC) using large volumes (≈6 mL) of the whole blood from venipuncture. Incessant blood drawings cause a great physical and psychological burden for cancer patients.^[^ ^11^ ^]^ In addition, CBC requires medical professionals and can only be performed in hospital or specialized health institutions with commercial hematology analyzers.^[^ ^12^ ^]^ Frequent hospital visits impact patient's quality of life and arise the financial burden. Furthermore, many patients are admitted to the hospital only when serious infections or fevers occur due to avoidance or omission of tests, making treatment more difficult.^[^ ^13^, ^14^, ^15^ ^]^ Therefore, an in situ and home‐use detection method is in urgent need to promptly reflect the neutrophil content of patients.

**Figure 1:**
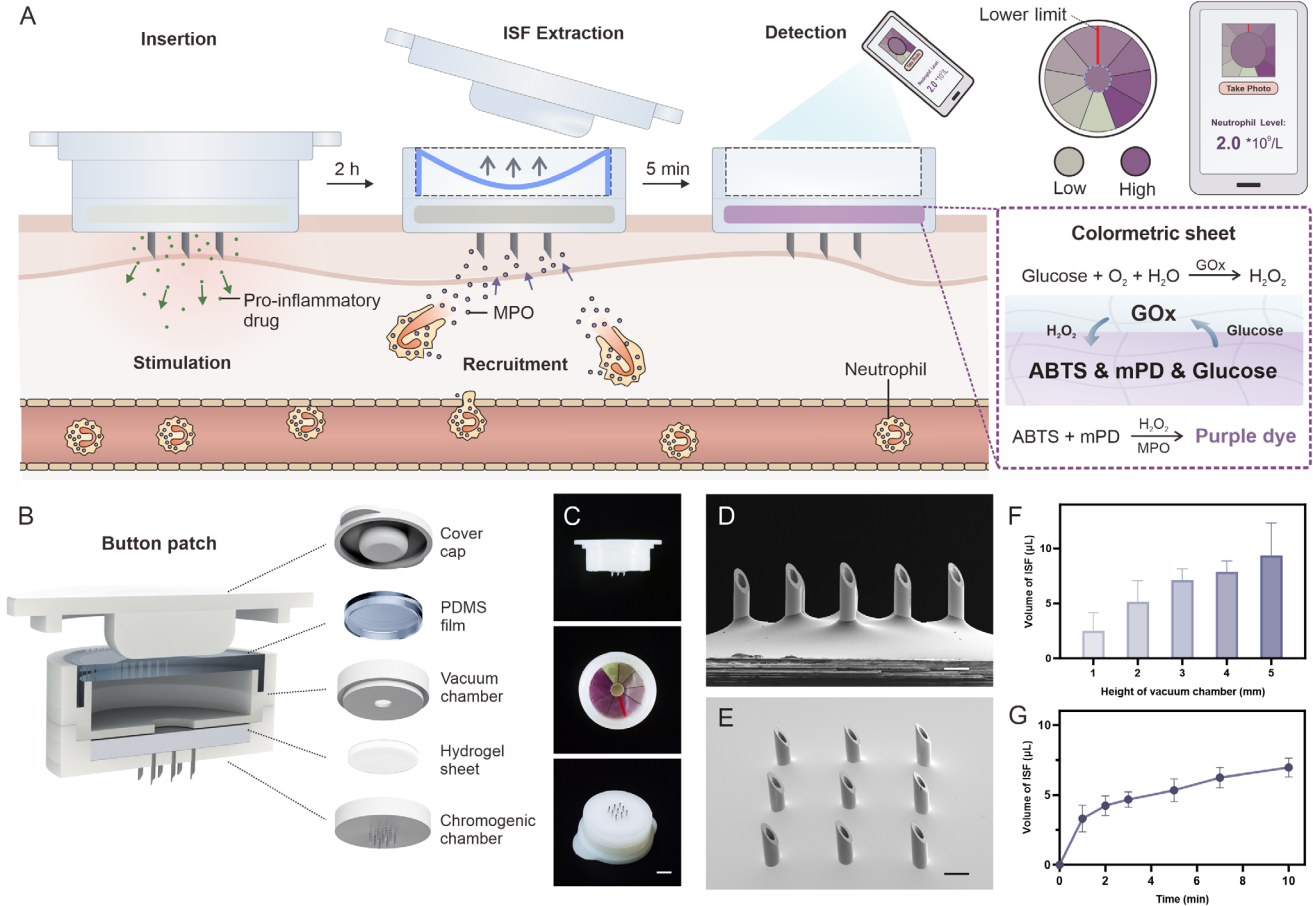
Schematic and characterization of the button patch for neutrophil detection. A) Schematic of the patch. After application onto the skin, blood neutrophils are locally recruited and release their specific myeloperoxidase into the ISF, which can be extracted onto a lyophilized hydrogel sheet for color development after the removal of the cap. Blood neutrophil levels can be understood by observing color development, and the data can be quantified through a mobile phone application. B) Structure of the patch. C) Photographs of side, top, and bottom views of the device. Scale bar: 3000 *µ*m. D, E) Scanning electron microscopy images of the microneedle array. Scale bar: 500 *µ*m. F) Comparison of the amount of ISF extracted by vacuum chambers of different heights (*n* = 3). G) Extraction profiles of 3 mm vacuum chamber (*n* = 3). In F and G, data are presented as mean ± SD.

Herein, we present a button‐like portable microneedle patch that can rapidly and easily detect neutrophil levels without vascular invasion (**Figure** **1**A). The bottom of the device is loaded with pro‐inflammatory substances (e.g., phorbol 12‐myristate 13‐acetate (PMA), histamine), which can create a local inflammatory microenvironment after insertion into the skin.^[^ ^16^, ^17^, ^18^, ^19^ ^]^ Upon stimulation, neutrophils are recruited and accumulated locally within hours, being the dominant players during acute inflammation.^[^ ^2^, ^20^ ^]^ Myeloperoxidase (MPO), a neutrophil‐specific enzyme, is subsequently released into the interstitial fluid (ISF), with a level positively correlated with blood neutrophil level.^[^ ^21^, ^22^, ^23^, ^24^ ^]^ Therefore, a vacuum‐mediated extraction system was integrated to achieve in situ collection of ISF through microneedle channels for further detection of MPO concentration. A visible color indicating the MPO level was displayed by the incorporated colorimetric sheet made of GelMA embedding 2,2’‐azino‐bis(3‐ethylbenzothiazoline‐6‐sulfonic acid) diammonium salt (ABTS), m‐phenylenediamine (mPD), and glucose in the bottom layer, and glucose oxidase (GOx) in the upper layer. Once contact with the MPO, the immobilized ABTS and mPD were catalyzed to produce a purple substance in the presence of hydrogen peroxide (H_2_O_2_) generated by glucose and GOx.^[^ ^25^ ^]^ This integrated device is portable, costless, and feasible for self‐operation by patients at home. Blood neutrophil levels can be visualized by the instant color display post‐application, avoiding the emotional and financial burden of frequent blood draws on cancer patients. Furthermore, a mobile phone application was developed for the simple quantification of the color development.

## 2 Results

### 2.1 Design and Fabrication of the Button Patch

The integrated system was assembled from five distinct subcomponents, which can be mainly divided into two parts: vacuum extraction system and colorimetric sensor (Figure 1B). The cover of the vacuum extraction system could be pre‐pressed with the polydimethylsiloxane (PDMS) soft film to create a negative pressure on demand (Figure 1C). Upon cover removal, ISF could be extracted through the microneedles into the hydrogel sheet in chromogenic chamber to start color development. An observation window with colorimetric card was integrated to the vacuum chamber to obtain half‐quantitative neutrophil levels. For in situ neutrophil recruitment upon skin application, pro‐inflammatory drugs such as PMA or histamine was loaded on the bottom of the device to induce a local inflammatory response (Figure S1, Supporting Information). Microneedles were arranged into a 3×3 array to extract a sufficient volume of ISF for detection (Figure 1D,E). The syringe needle‐like structure was shown in the scanning electron microscope (SEM) images with an outer diameter of 300 *µm*, an inner diameter of 150 *µm*, a height of 950 *µm*, and a spacing of 1200 *µm* (Figure S2, Supporting Information). The mechanical test verified sufficient mechanical strength of the microneedle array to penetrate skin (Figure S3, Supporting Information).

The extraction of ISF using the button patch was examinated with vacuum chambers of different sizes (6 mm in radius, and 1–5 mm in height). As shown in Figure 1F, the extraction volume increased in accord with the chamber volume. Among them, chambers with heights of 3 mm and 4 mm achieved sufficient extraction volume and stable extraction efficiency, and 6 mm in radius with 3 mm in height was determined as the dimensions of the chamber for the following experiments. The patch drew ≈5 *µL* of ISF from rat skin within 5 minutes, followed by a gradual increase (Figure 1G).

### 2.2 The Performance of Button Patch In Vitro

**Figure 2:**
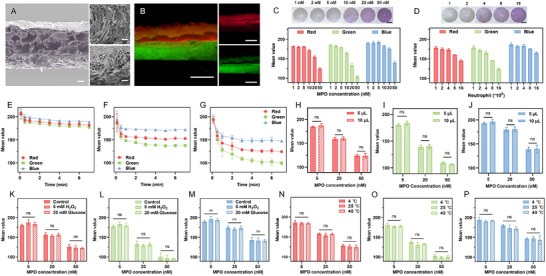
Characterizations of the lyophilized hydrogel sheet. A) Scanning electron microscopy images of the double‐layered hydrogel sheet. Left scale bar: 200 *µ*m. Right scale bar: 20 *µ*m. B) Fluorescence microscopy images of the cross‐section of the hydrogel sheet containing Cy5‐labeled GOx and FITC in represents of ABTS and mPD. Scale bar: 1000 *µ*m. C) Up: Photographs showing the color changes of the hydrogel sheet after MPO solution (1‐50 nM) absorption for 5 minutes. Scale bar: 1000 *µ*m. Down: RGB mean values of the circular observation window in the taken picture (*n* = 3). D) Up: Photographs showing the color changes of the hydrogel sheet after PMA‐stimulated neutrophil cultures (1‐16 ×10^6^/mL) absorption for 5 minutes. Scale bar: 1000 *µ*m. Down: RGB mean values of the circular observation window in the taken picture (*n* = 3). E–G) Color development profile of the hydrogel sheet added with different concentrations (low: 5 nM, middle: 20 nM, high: 50 nM) of MPO solution (*n* = 3). H–J) RGB mean values of the photograph of hydrogel sheet added with different concentrations (low: 5 nM, middle: 20 nM, high: 50 nM) of MPO solution at different volumes (*n* = 3). K–M) RGB mean values of the photograph of hydrogel sheet added with different concentrations (low: 5 nM, middle: 20 nM, high: 50 nM) of MPO solution with the presence of 5 mM H_2_O_2_ and 20 mM glucose (*n* = 3). N–P) RGB mean values of the photograph of hydrogel sheet added with different concentrations (low: 5 nM, middle: 20 nM, high: 50 nM) of MPO solution at different temperatures (*n* = 3). In C‐P, data are presented as mean ± SD. In H‐J, statistical significance was determined by the two‐tailed Student's t‐test. In K‐P, statistical significance was determined by the one‐way ANOVA. *P < 0.05, **P < 0.01, ***P < 0.001, ****P < 0.0001.

To achieve in situ detection of neutrophil level, a double layered lyophilized GelMA hydrogel sheet was prepared for quick ISF absorption and uniform color development. The bottom layer contained ABTS and mPD as chromogenic molecules, and glucose which could act with GOx in the upper layer to generate H_2_O_2_ in situ to launch color development in the presence of MPO. The methacrylation of GelMA was confirmed by proton nuclear magnetic resonance (^1^H‐NMR) spectra, and the methacrylation degree was ≈30% (Figure S4, Supporting Information). The porous and stacked structure of the hydrogel sheet was observed from the SEM image (**Figure** **2**A). The porous structure in the dry state ensured a strong water absorption capacity while the gel state could prevent the diffusion of internal molecules after absorbing water, avoiding the “coffee ring” effect during color development (Figure S5, Supporting Information). The fluorescence microscopy images presented the distribution of the Cy5‐labeled GOx in the top layer and FITC in the bottom layer representing ABTS and mPD (Figure 2B). Once ISF enters the hydrogel, the glucose in the bottom layer will be dissolved and encounter the GOx in the upper layer, generating H_2_O_2_ to initiate the color development reaction. The chromogenic substrate is evenly distributed in the horizontal direction of the hydrogel sheet, ensuring uniform color development (Figure S6, Supporting Information). To determine the in vitro color readout of the lyophilized hydrogel sheet in response to different MPO levels, 5 *µ*L of MPO solution were added to the bottom of the hydrogel sheet. Photos were taken for RGB analysis after absorbing for 5 minutes. With the increase of MPO level, the RGB value gradually dropped with a changing rate of Green > Red > Blue, resulting in a more purple color to the naked eye (Figure 2C). Good linearity was shown in the quantitative analysis of green colors in the range of 1‐20 nM MPO (R^2^ = 0.9755, Figure S7, Supporting Information). Subsequently, we examined the relationship between neutrophils and color development in vitro. PMA‐stimulated neutrophil cultures were added to the hydrogel sheet for color development. Consistent with the MPO results, the hydrogel sheets achieved gradient color development for neutrophil content with an R‐squared of 0.9630 (Figure 2D; Figure S8, Supporting Information).

Further, we verified the stability of the color development system in terms of reaction time, extraction volume, biomolecules, and temperature. The system reacted rapidly within the first minute of MPO contact, and the color change gradually slowed down and stopped after 5 minutes (Figure 2E–G). Then we examinated whether the volume of the extracted ISF would affect the results of the color development. The increase of the liquid volume from 5 *µL* to 10 *µL* did not change colorimetric results (Figure 2H–J). Glucose and H_2_O_2_ are two common biomolecules involved in the color development reaction. ISF contains glucose at the mM level,^[^ ^26^ ^]^ and the concentration of H_2_O_2_ at the site of inflammation has been reported as high as 1 mM.^[^ ^27^ ^]^ Accordingly, high concentration of glucose was added to the system to reduce the error and the color development was not affected by the addition of 5 mM H_2_O_2_ or 20 mM glucose (Figure 2K–M). Moreover, we demonstrated that the cold environment (4 °C), room temperature (25 °C), and body temperature at fever (40 °C) had negligible effect on the color development results (Figure 2N–P). Finally, placing the sealed hydrogel sheet at room temperature (25 °C) for one month did not affect its colorimetric ability, demonstrating the long‐term stability of the device (Figure S9, Supporting Information).

### 2.3 Detection of Neutrophil Levels in a Neutropenic Rat Model

**Figure 3:**
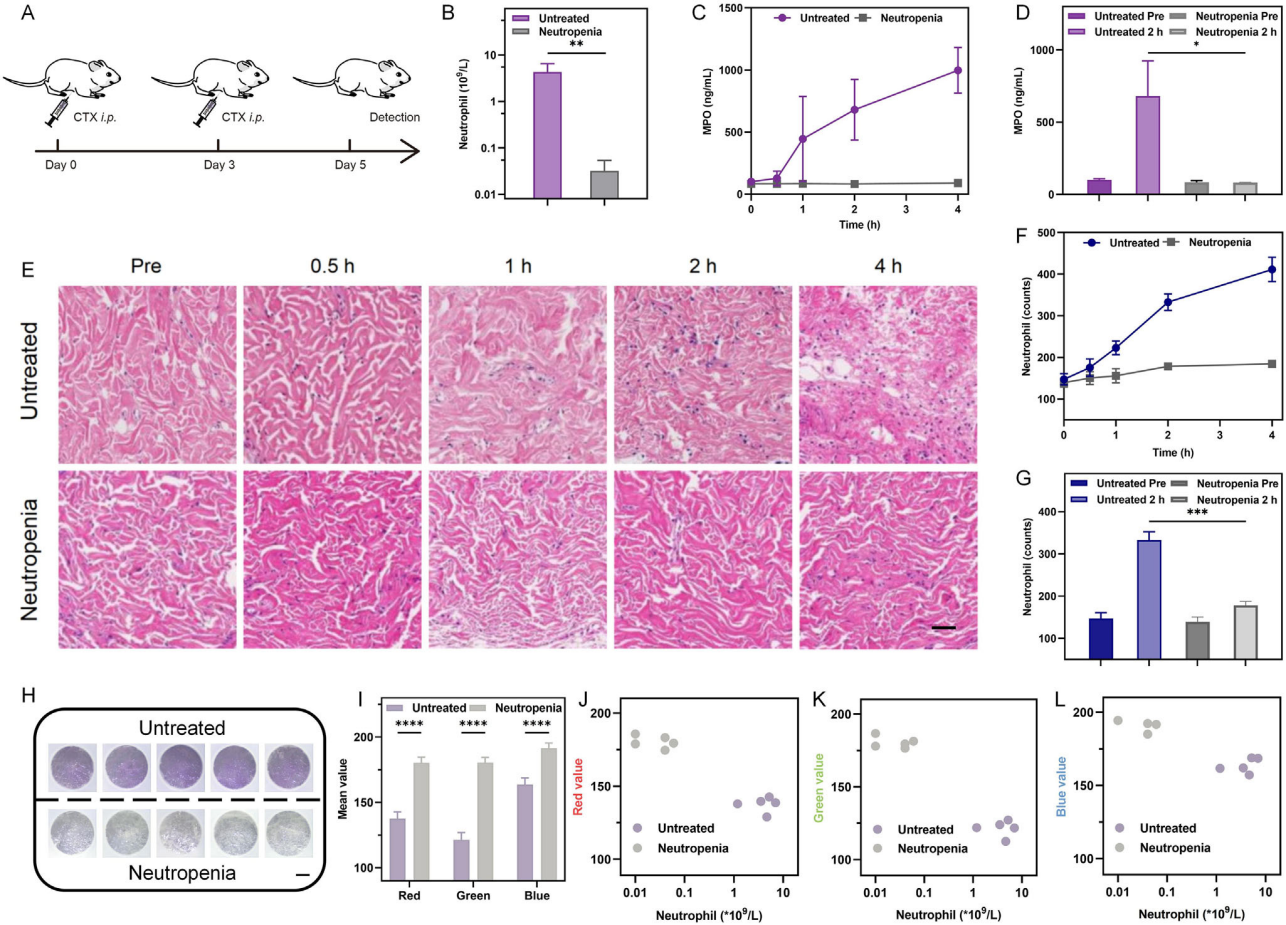
Comparison of MPO and neutrophil levels of untreated and neutropenia rats. A) Schematic of the induction of rat neutropenia model and the timing of detection. B) Comparison of blood neutrophil levels of untreated and neutropenia rats at day 5 (*n* = 5). C) ISF MPO level of untreated and neutropenia rats at different time points after the onset of inflammatory stimulation (*n* = 3). D) Comparison of ISF MPO level at 2 hours after the onset of stimulation (*n* = 3). E) Representative hematoxylin and eosin (H&E) stained sections of rat skin before and after pro‐inflammation. Scale bar: 1 mm. F) Quantification of skin neutrophil level from H&E stained sections of rat skin (*n* = 3). G) Comparison of skin neutrophil level at 2 hours after the onset of stimulation (*n* = 3). H) Photographs of the hydrogel sheet within the observation window after 2 hours of patch application with PMA and 5 minutes of ISF extraction. Scale bar: 1000 *µ*m. I) RGB mean values of the circular observation window in the picture in H) (*n* = 5). J–L, Scatter plot showing the colorimetric results on each rat corresponding to blood neutrophil levels (*n* = 10). In B–D, F, G, and I, data are presented as mean ± SD. Statistical significance was determined by the two‐tailed Student's t‐test. *P < 0.05, **P < 0.01, ***P < 0.001, ****P < 0.0001.

Cyclophosphamide‐induced neutropenic rat model was established to investigate the in vivo performance of the device (**Figure** **3**A). The neutrophil levels dropped below 100 cells/mm^3^ at day 5 (Figure 3B) and microneedle device containing 100 *µg* PMA was applied to the dorsal skin of rats to induce mild local inflammation. As shown in Figure 3C, the concentrations of MPO in normal rats gradually increased with the development of inflammation but remained essentially unchanged in neutropenic rats. A significant difference of 8.3‐fold was observed 2 hours post stimulation (Figure 3D). Hematoxylin and eosin (H&E) staining was subsequently conducted to evaluate the neutrophil recruitment after inflammatory stimulation. Neutrophils were locally enriched in normal rat skin within half an hour after the local stimulation, and significantly increased at 2 hours. However, the number of neutrophils in the skin of neutropenic rats showed a negligible change, which is consistent with the MPO results (Figure 3E–G; Figure S10, Supporting Information). The fold‐change of neutrophils was less than the change in MPO (1.9 vs 8.3), which can be due to impaired function of residual neutrophils after chemotherapy.^[^ ^28^ ^]^

The in vivo performance of the integrated button patch was demonstrated in neutropenic rats. As a direct agonist of neutrophils, PMA was first selected as a pro‐inflammatory drug for experimentation. The patch was inserted into the rat's dorsal skin and kept for 2 hours before the cap was removed to start ISF extraction of 5 minutes. The initial white sheet started to develop a purple coloration in the normal rats but remained white in the neutropenic rats (Figure 3H and Videos S1, S2, Supporting Information). The picture from the observation window also exhibited a notable decrease in RGB mean values (Figure 3I–L). In addition, we established a rat acute inflammation model by *i.p*. injection of LPS and used the microneedle device with halfed concentration of the colorimetric substrate for detection. Figure S11 (Supporting Information) showed that the colorimetric depth in inflamed rats was significantly higher than that in untreated rats. Subsequently, we examined the recovery of the skin after device removal. The topical skin was washed with water after the device removal to clear the potentially residual PMA. The micro‐sized channels gradually disappeared within 1 hour, and no obvious inflammatory manifestations were seen after 12 hours (Figure S12, Supporting Information). Furthermore, histamine, an FDA approved skin prick test drug, was also examined as a pro‐inflammatory substance for local stimulation.^[^ ^29^ ^]^ Similar results were detected in both healthy rats and neutropenic rats after a 4‐hour stimulation (Figures S13 and S14, Supporting Information), and no visible signs of skin inflammation can be seen 12 hours after device removal (Figure S15, Supporting Information). Consistent with the photos, no significant neutrophil infiltration was observed in the skin 24 hours after the removal of the PMA or histamine‐loaded device (Figures S16 and S17, Supporting Information). Moreover, the application of the devices did not significantly influence the blood neutrophil and leukocyte counts (Figure S18, Supporting Information).

### 2.4 Clinical Applications of the Button Patch

Clinically, in the postoperative period or in case of infection, neutrophil level in local biofluid directly reflects the severity of inflammation. For instance, patients with urinary tract infections often have a large number of neutrophils in urine, and regular urinalysis is required for the inflammation assessment. Similarly, patients undergoing abdominal surgery often have drains placed in the abdominal cavity to drain the peritoneal fluid after surgery.

**Figure 4:**
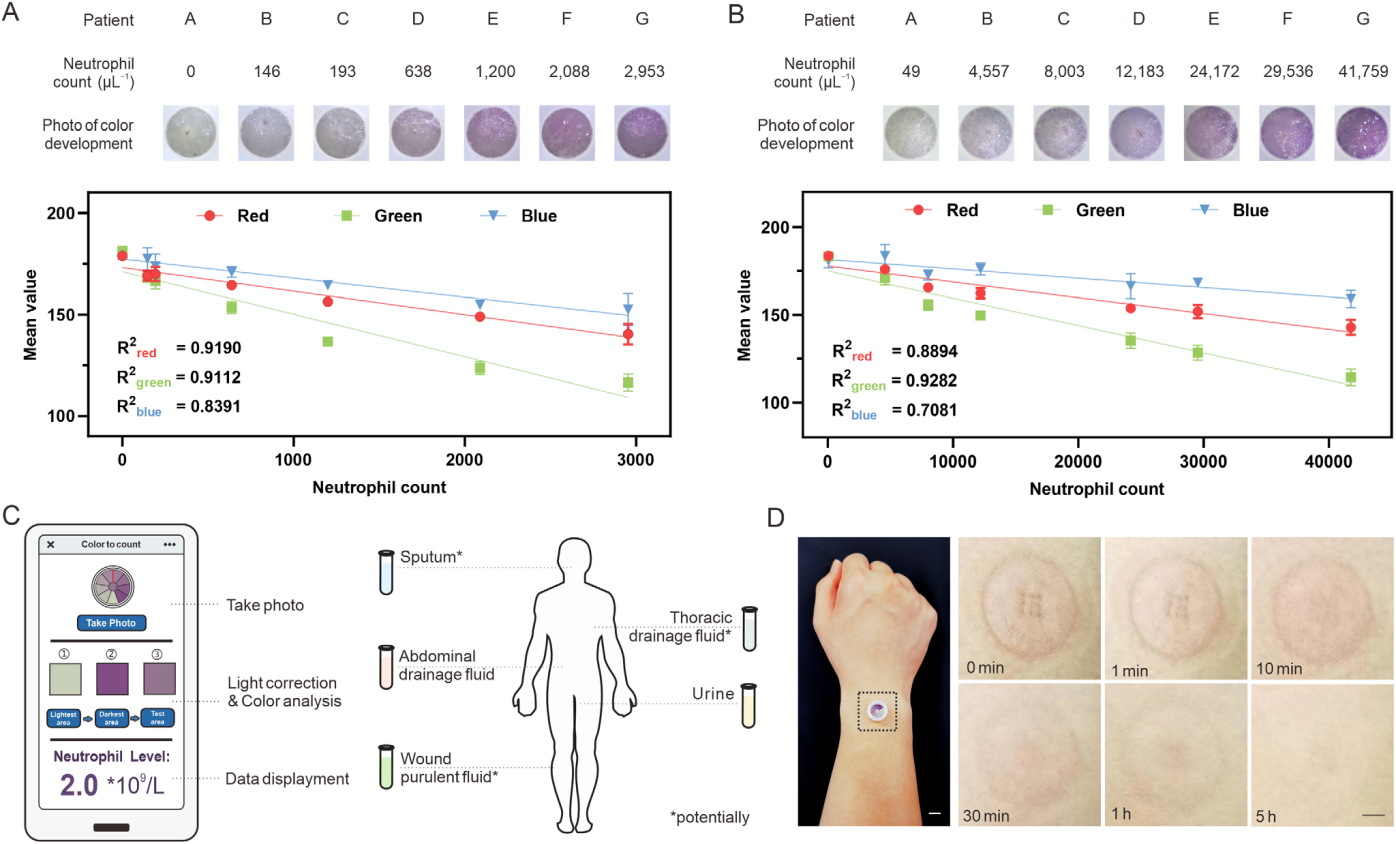
Clinical translation potential of the button patch. A, B) Neutrophil counts of patient's urine A) and abdominal drainage fluid B) samples with corresponding color developed images. Scale bar: 1000 *µ*m. And linear regression of the red, green, and blue values against neutrophil concentration in human abdominal drainage fluid. C) Demonstration of the software interface and illustration of the application potential of the patch clinical body fluid samples. D) Demonstration of the patch application (left) and photographs of human skin recovery at different times after patch removal (right). Left scale bar: 1 cm. Right Scale bar: 3000 *µ*m. In A and B, data are presented as mean ± SD.

With a sample requirement of only 5 *µL*, the button patch can realize quick detection of a wide range of body fluids commonly found in clinical settings such as sputum, thoracic or abdominal drainage fluid, urine, and wound purulent fluid. Human urine and abdominal drainage fluid were collected from participants as representative samples. Due to the low neutrophil content in urine, the substrate concentration was doubled in the hydrogel sheet. As shown in **Figure** **4**A, significant color development occurred in all the inflammatory urine samples, reaching a good linear relationship (R^2^ = 0.9112) between the color development and the sample neutrophil count. Additionally, we tested the color development of abdominal drainage fluid in 7 postoperative laparoscopic cholecystectomy patients. The patch can achieve gradient color development for neutrophils in the abdominal drainage fluid (R^2^ = 0.9282, Figure 4B).

To quantify the color development data, a mobile application was developed to analyze the color development. After the completion of color development, the PDMS soft film can be easily removed to take a photograph with a mobile phone. A color correction procedure is first conducted by selecting the lightest and the darkest area of the colorimetric card to eliminate the effects of different lighting conditions at the time of shooting. Subsequently, color analysis of the chromogenic area can be performed to read out the patients' neutrophil value (Figure 4C). During clinical application, doctors can make initial evaluations by looking at the color and accurate judgments can be done through mobile application when essential.

We also assessed the on‐body safety of the device to validate the clinical translatability. The patch was lanced into the subject's skin after ethylene oxide sterilization and held for 2 hours before removal to observe the skin recovery. The micro‐sized holes healed quickly within 10 minutes, followed by a slight reddening of the skin fully recovered within 5 hours (Figure 4D). There was no obvious pain throughout the process, demonstrating the good human safety of the device.

## 3 Conclusion

In the last decade, microneedle device has been extensively explored for the detection of various biomarkers.^[^ ^30^ ^]^ Nonetheless, most measurements are limited to ions (e.g., Na^+^, K^+^),^[^ ^31^ ^]^ small molecules (e.g., glucose, antibiotics,^[^ ^32^ ^]^ or large biomolecules (e.g., protein, DNA).^[^ ^33^ ^]^ Few studies reported the non‐invasive detection of cells.^[^ ^34^ ^]^ To this end, we developed a colorimetric device for rapid in situ detection of neutropenia. As a widely used neutrophil marker, the local concentration of MPO is highly correlated with the concentration of neutrophils and is therefore selected as a target for detection. In our design, a vacuum‐based extraction system was integrated to ensure a sufficient volume of ISF. The protuberant cover enables the vacuum to be pre‐stored in the device and can be removed on demand to initiate extraction of the ISF. Uniform and stable presentation is the essential point in colorimetric reaction. Several polymeric stabilizers have been used to immobilize the chromogenic substrates (e.g., polyethyleneimine, *β*‐cyclodextrin).^[^ ^35^, ^36^ ^]^ However, this can greatly reduce the sensitivity of the chromogenic development. In the present work, we prepared a lyophilized cross‐linked hydrogel sheet possessing highly porous structure and immobilized the substrates to avoid the “coffee ring” effect. Moreover, this hydrogel sheet demonstrated strong color development stability against varying of extraction volume, biomolecules, and temperature.

In summary, this button patch could greatly reduce the cost and technical threshold of blood testing, holding promising implications for public health in underdeveloped regions. In addition to intuitive semi‐quantitative detection based on colorimetric cards, the accompanying mobile application can achieve quantitative analysis of neutrophil levels. In the future, patient's data can be uploaded to hospital databases through applications, facilitating real‐time monitoring of homebound patients by physicians.

## 4 Experimental Section

### Materials

All the chemicals were purchased from Aladdin unless otherwise specified or were used as received. PDMS prepolymers and curing agent (Sylgard 184 Silicone Elastomer) were purchased from Dow Corning (MI, USA). Type A gelatin from porcine skin was purchased from Beyotime. The photoinitiator Irgacure 2959 was purchased from Sigma‐Aldrich. Rat blood neutrophil separation kit was purchased from Solarbio. Rat neutrophil ELISA kit was purchased from Develop.

### Fabrication of the Microneedle Device

The hollow stainless‐steel microneedles were fabricated using laser cutting. The polyformaldehyde extraction chamber and the vacuum chamber were designed using SOLIDWORKS and fabricated by machining. For the fabrication of the PDMS soft film, the prepolymer base Sylgard 184A and curing agent Sylgard 184B were thoroughly mixed in a 10:1 weight ratio. The mixture was poured onto the molds and cured at 80 °C for 3 h. The four parts were carefully assembled and sealed with resin adhesive.

### Mechanical Strength of Microneedle

The mechanical property was tested by compression testing machine (XinSanSi Shanghai enterprise development Co., Ltd., China) by pressing the upright single needle against a stainless‐steel plate. The force‐displacement curve of the single needle fixed on the bottom of the device was recorded.

### Preparation of GelMA

5 g of gelatin was dissolved in 50 mL of DPBS under constant stirring at 50 °C. 4 mL of methacrylic anhydride was added dropwise into the solution under vigorous stirring and reacted for 3 h at 50 °C. Afterward, 200 mL of DPBS (50 °C) was added to stop the reaction. The solution was dialyzed using 8‐10 kDa membrane for a week to remove the residual methacrylic anhydride. The GelMA prepolymer was obtained though lyophilization at ‐80 °C.

### Preparation of the Hydrogel Sheet

0.18 g glucose, 0.5 g GelMA and 0.05 g photoinitiator were dissolved in 10 mL DI water at 60 °C to prepare solution A. 49.38 mg ABTS and 9.73 mg mPD were dissolved in 18 mL DI water at room temperature to prepare solution B. 0.25 g GelMA and 0.025 g photoinitiator were dissolved in 5 mL DI water at 60 °C and 20 mg of GOX was added at 37 °C to prepare solution C. 125 *µ**L*** of solution A was casted onto the PDMS mold and was exposed to 500 mW cm^−2^ UV light (360‐480 nm) for 150 s. Following the exposure, the filled mold was soaked into solution B for two hours. After removal of solution B, 50 *µL* of solution C was casted onto the mold to cover the hydrogel sheet. Subsequently, the filled mold was kept at ‐80 °C for 12 h before being subjected to a freeze dryer.

### Comparison of Color Presentation on Different Carriers

Purple dye was made by mixing ABTS, mPD, MPO, and H_2_O_2_ together and reacting for 1 h until the termination of chromogenic reaction. Then, 5 *µL* of the purple solution was added to the nitrocellulose membrane or GelMA sheet representatively. Photos were taken after 2 min to record color presentation.

### Animal Model

Female, Sprague‐Dawley rats weighing 150–200 g were used in the experiments. For rat neutropenia model, cyclophosphamide was given intraperitoneally at 150 mg/kg at day 0 and 50 mg/kg at day 3. Animals were monitored for evidence of overt illness, and blood samples were taken at day 5 to identify their neutrophil levels. For rat inflammation model, LPS was given *i.p*. at dose of 10 mg/kg and blood samples were taken at 12 h after LPS injection.

### In Vitro Test Using Rat Neutrophil

Neutrophil was isolated from anticoagulated rat whole blood according to the given protocol of rat blood neutrophil separation kit. Subsequently, neutrophils were resuspended at different concentrations and 1 *µ*M PMA were added for 2 hours stimulation. The supernatant was obtained by centrifugation at 300 g for 10 min and added to the hydrogel sheet for color development.

### In Vivo Studies

All animal experiments were performed according to an animal study protocol approved by the Institutional Animal Care and Use Committee at Zhejiang University (ZJU20220348). The rats were shaved one day before the microneedle device application. To determine the MPO content in the ISF of rat skin after application of the microneedle device, the microneedle was removed two hours after application and local aspiration was performed using negative pressure for 10 min. The extracted ISF was collected and MPO content was measured using ELISA kits. To evaluate the sensing ability of the microneedle device, blood neutrophil level of the rat was first recorded using routine blood test through the orbital venous plexus blood (≈100 *µL*). During the application of the device, the rats were anesthetized with isoflurane to avoid movement. Microneedle device with 100 *µg* PMA was firmly pressed for the initial 10 s to fully attach and penetrate the skin. After 2 h, the plastic cover was removed to launch ISF extraction. Color change would occur within the next 5 min and the device was removed before the photograph was taken to record the color change of the hydrogel sheet. When the pro‐inflammatory substance was replaced with 500 *µg* histamine, the retention time of the device on the skin was extended to 4 hours without changing other conditions. For linearity experiment, microneedle patch was applied to neutrophilia rat dorsal skin 12 h after LPS injection (10 mg/kg), and blood sample was taken at the same time for comparison. Neutropenia rats were established by cyclophosphamide *i.p*. injection at dose of 150 mg/kg, microneedle patch was applied to the rat skin at day 0, day 3, day 6, and day 9 with blood sample taken at the same time for comparison.

### Detection Experiments with Human Samples

Informed consent was obtained from all participants prior to the commencement of any human experiments. All the experiments were conducted strictly in accordance with the protocol approved by Medical Ethics Committee of Sir Run Run Shaw Hospital Affiliated to Zhejiang University School of Medicine (SRRSH20230531 and SRRSH20230556). Abdominal drainage fluid and urine samples were retrieved from the biospecimen bank at Sir Run Run Shaw Hospital and stored at ‐80 °C. Due to the high neutrophil content, the peritoneal drainage fluid was diluted 5 times before used for the colorimetric experiment. For the color development experiments, 5 *µ*L of liquid was added to the PDMS sample tank, after which the microneedle device was placed on the sample tank and extraction was initiated. After 5 minutes, the device was taken out, the PDMS elastomer membrane was removed, and photographs were taken to record the color development. Each sample was tested in triplicate.

### RGB and Analysis

The photo of the hydrogel sheet was taken by HD digital microscope camera under standardized light condition. The image was analyzed using ImageJ.

### Statistical Analysis

Statistical analyses were performed using GraphPad Prism 9.3.1. One‐way ANOVA was performed for multiple comparisons. Unpaired two‐tailed Student's t‐test was used for two group comparisons. *P* < 0.05 were defined statistically significant. The sample size used for statistical analysis was 3–5 according to experimental requirements. Data were presented as mean ± SD.

## Conflict of Interest

Z.G., Y.Z., J.C.Y., M.C., Z.L., and X.Y. have applied for a patent related to this work. Z.G. is the co‐founder of Zenomics Inc. and ZCapsule Inc. Z.G. and Y.Z. are the co‐founders of *µ*Zen Pharma Co., Ltd., and the other authors declare no conflict of interest.

## Author Contributions

M.C., Y.Z., Z.G., X.C., and Z.L. proposed the conception of the project. Z.L., R.C., T.X., S.Z., S.J., W.T., J.C.C., T.S., W.Z., H.W., J.H.Y., Y.K.S., Y.C.S., R.S., Z.Z., K.J., Z.Q.G., and X.Y. performed all the experiments and collected the data. T.X., S.L., J.C.C., J.H., and T.C. collected the clinical samples. Z.L., R.C., Y.S., J.C.Y., Y.Z., and M.C. prepared the manuscript. All the authors discussed the results and commented on the manuscript.

## Supporting information



Supporting Information

Supplementary Video S1

Supplementary Video S2

## Data Availability

The data that support the findings of this study are available in the supplementary material of this article.
