# Supplementary material for: Microneedle Patch for In Situ Neutrophil Monitoring
Source: Adv Sci (Weinh). 2025 May 8;12(23):2417801. doi: 10.1002/advs.202417801 (PMC12199443; doi:10.1002/advs.202417801)
Supplement: Supplementary file 1 — Supporting Information [file ADVS-12-2417801-s002.docx]

Supporting Information

Microneedle Patch for *in situ* Neutrophil Monitoring

*Ziyi Lu, Ruisi Cai, Tianao Xie, Sheng Zhao, Sarun Juengpanich, Win Topatana, Shijie Li, Jiasheng Cao, Jiahao Hu, Tianen Chen, Jiachen Chen, Jicheng Yu, Tao Sheng, Wentao Zhang, Hao Wang, Jiahuan You, Yukai Shan, Yuchao Sun, Ruijing Shen, Zhengjie Zhao, Kangfan Ji, Ziqi Gao, Xinmin Yu, Xiujun Cai^*^, Zhen Gu^*^, Yuqi Zhang^*^, Mingyu Chen^*^*


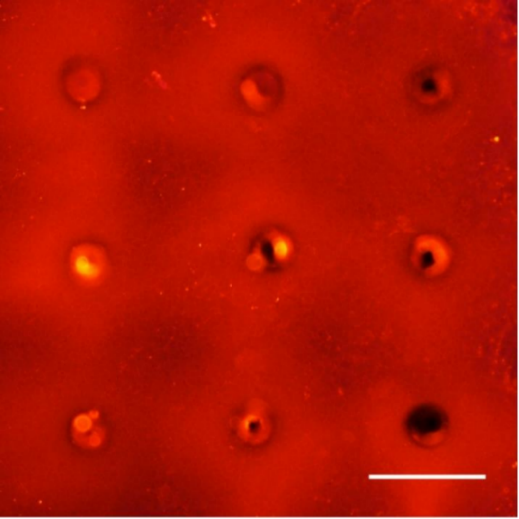


**Figure S1** Fluorescence microscopy images of the bottom of the patch loaded with rhodamine B representing pro-inflammatory drugs. Scale bar: 1 mm.


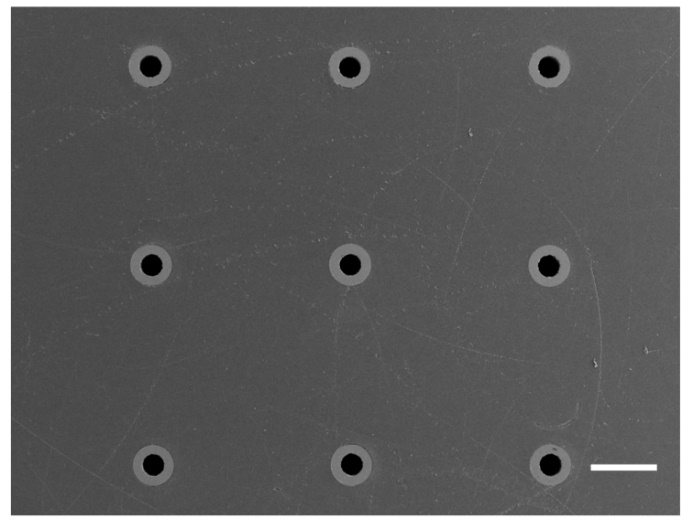


**Figure S2** Scanning electron microscopy image of the bottom view of the microneedle array. Scale bar: 500 *µ*m.


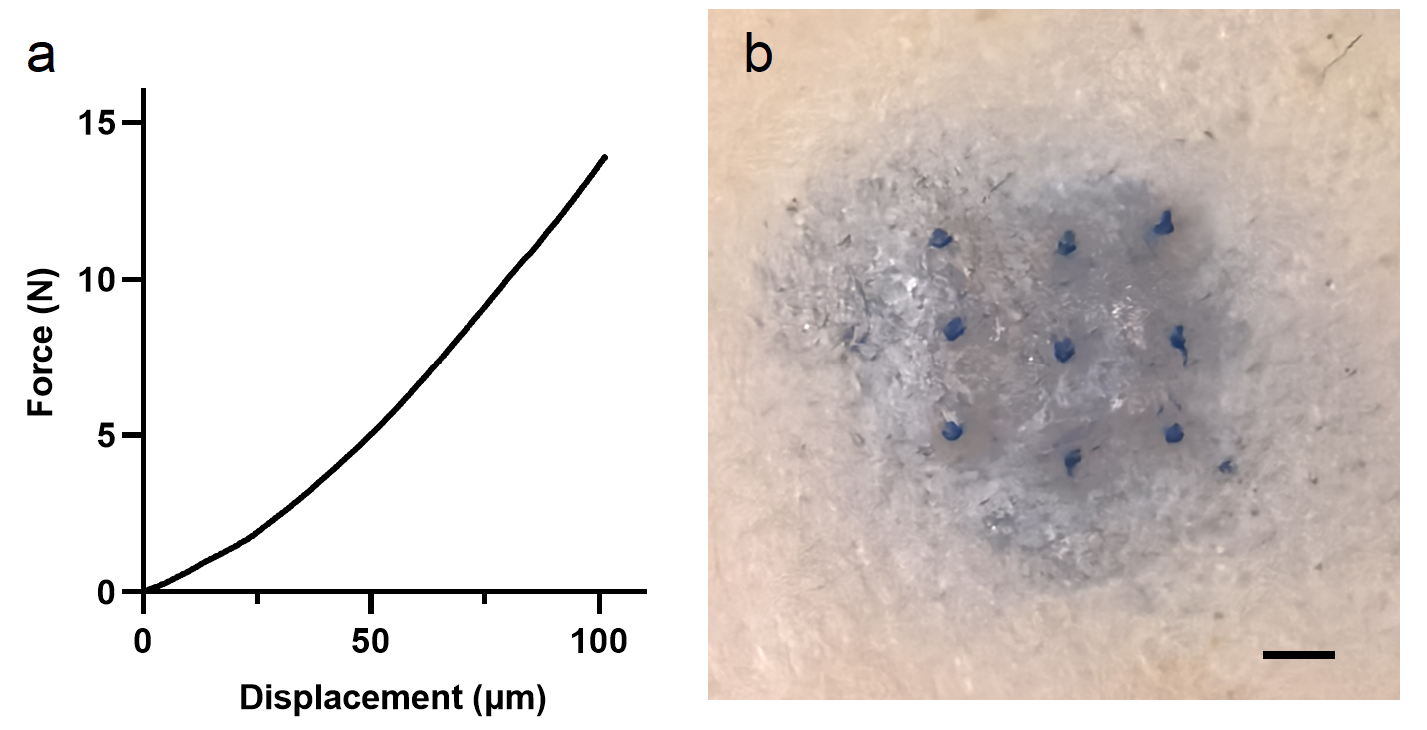


**Figure S3** The mechanical behavior of microneedles. (a) The force-displacement curve of a single microneedle. (b) Trypan blue staining of rat skin after the application of microneedles. Scale bar: 1000 *µ*m.


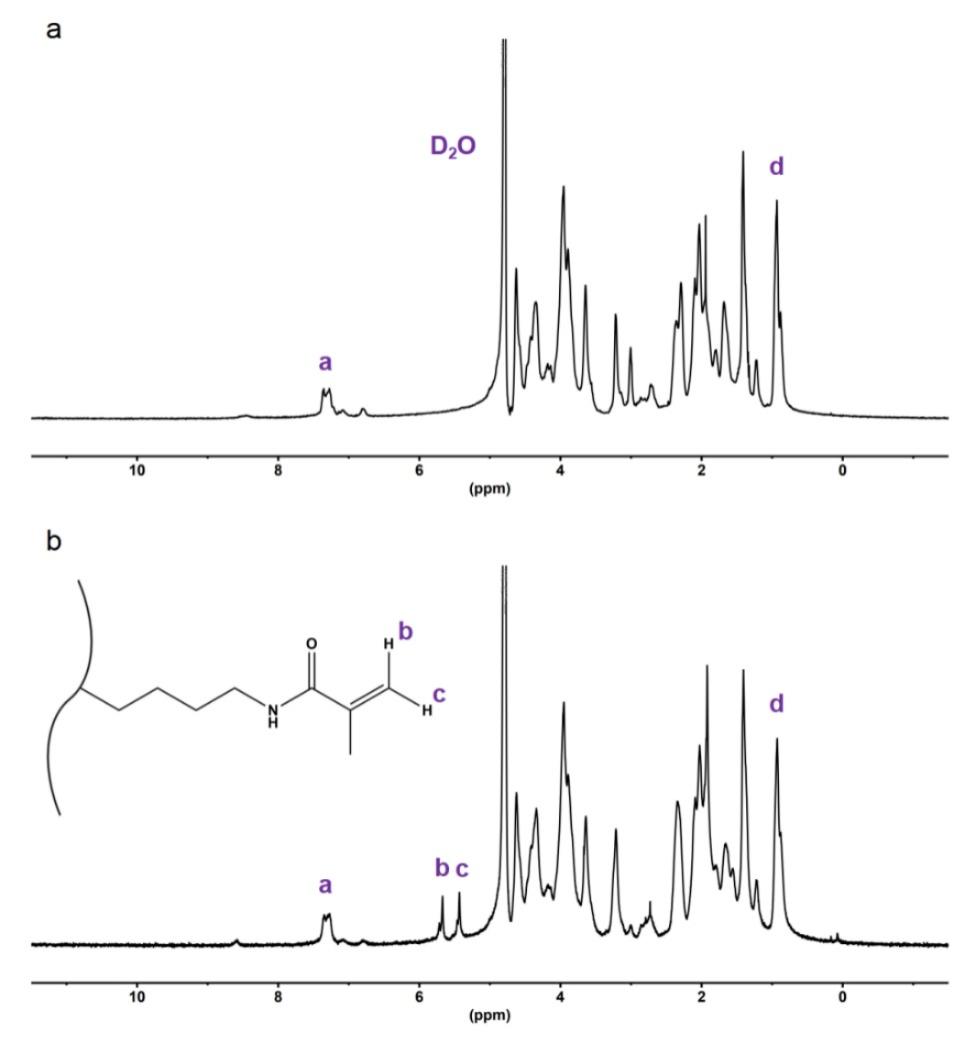


**Figure S4** ^1^H-NMR spectra of gelatin (a) and GelMA (b) in D_2_O. The peak a was attribute to aromatic residues. The characteristic peak b and c were due to the incorporation of vinyl groups of methacrylate into gelatin. The peak d was ascribed to the side chains of valine, leucine, and isoleucine.


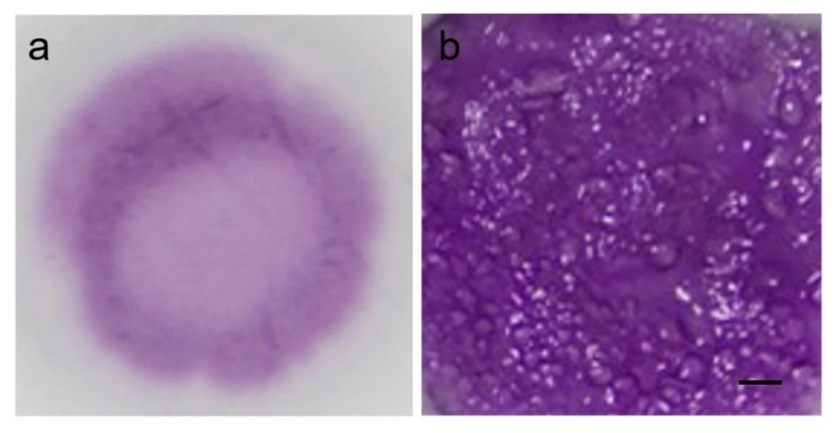


**Figure S5** Color presentation on nitrocellulose membrane (a) and GelMA sheet (b). Scale bar: 200 *µ*m.


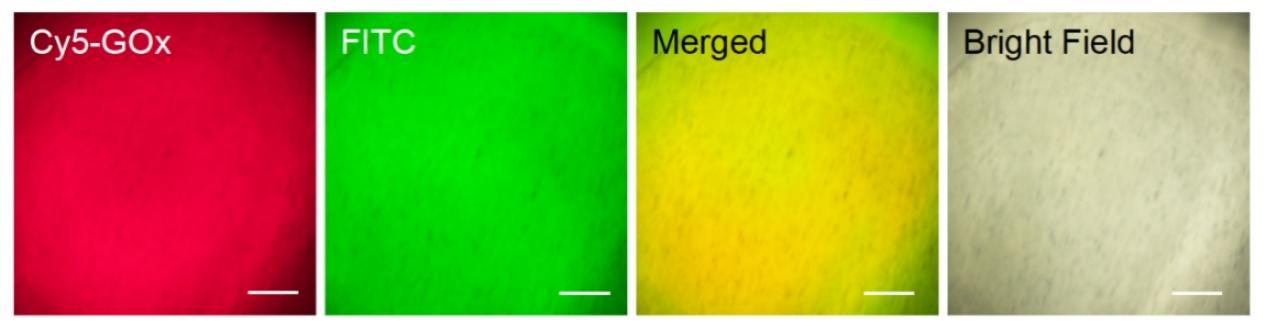


**Figure S6** Fluorescence microscopy images of the top view of the hydrogel sheet containing Cy5-labled GOx and FITC in represent of ABTS and mPD. Scale bar: 1000 *µ*m.


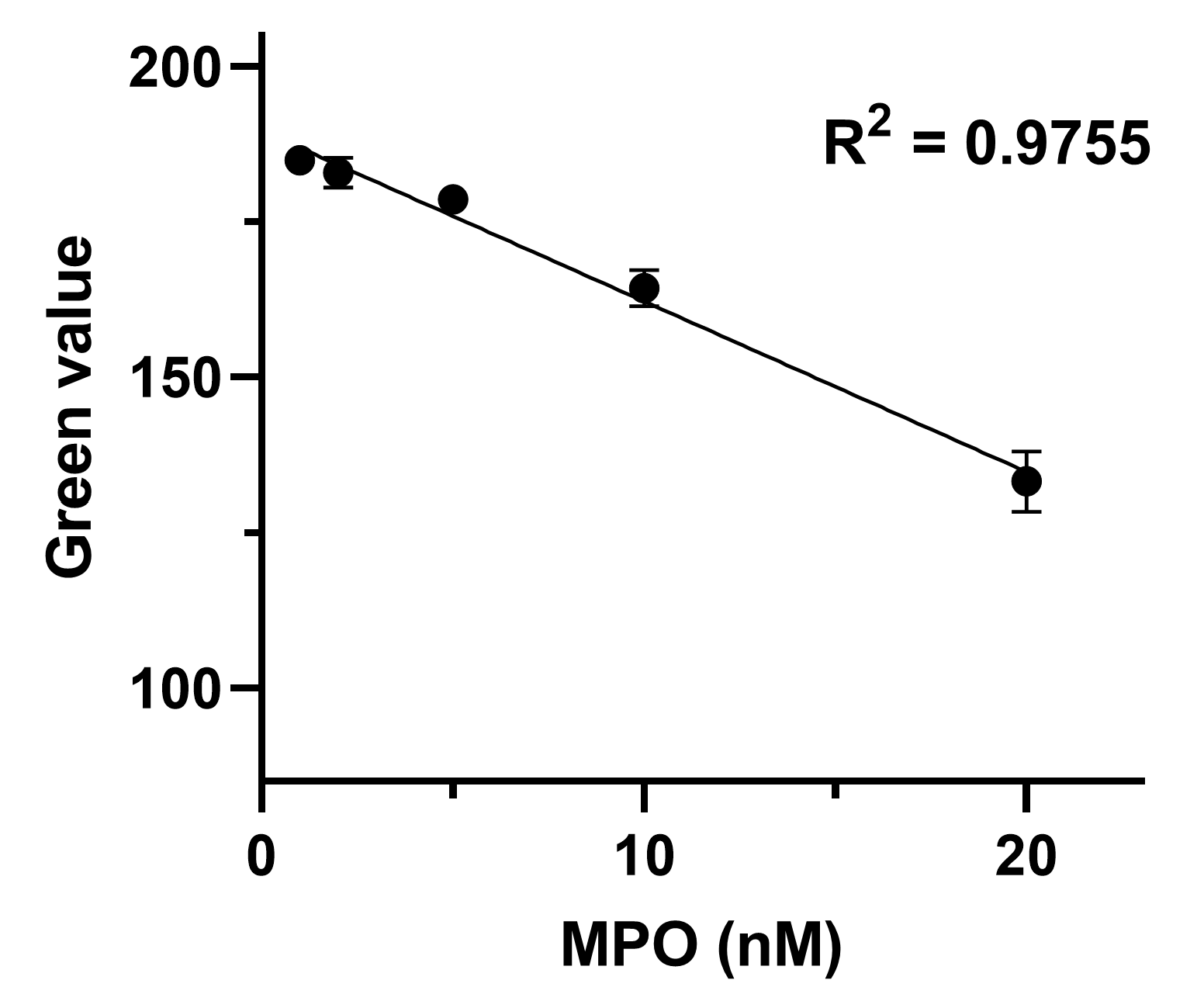


**Figure S7** Linear regression of the green values against MPO concentration (1-20 nM). Data are presented as mean ± s.d..


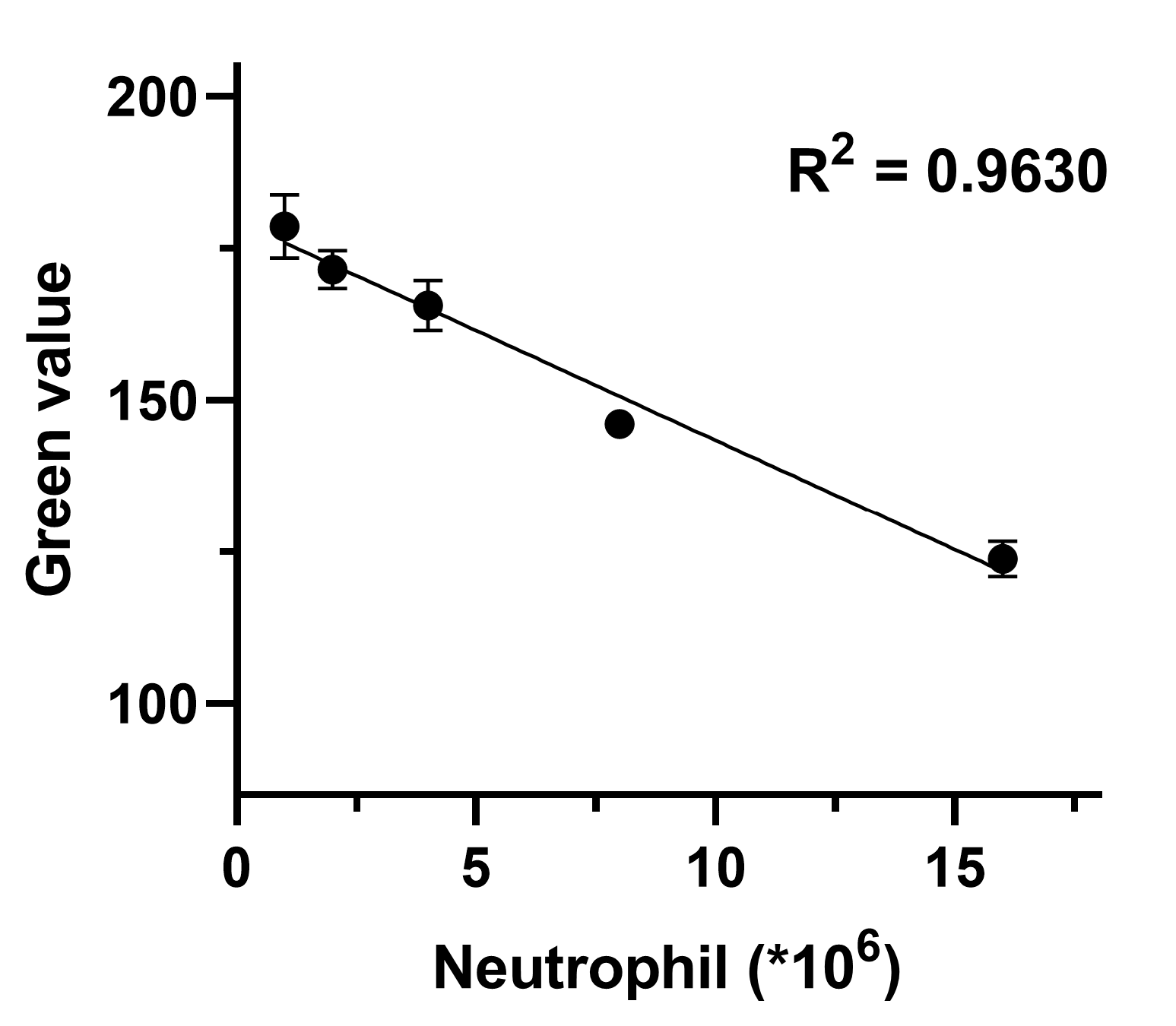


**Figure S8** Linear regression of the green values against neutrophil counts (1-16*10^6^). Data are presented as mean ± s.d..


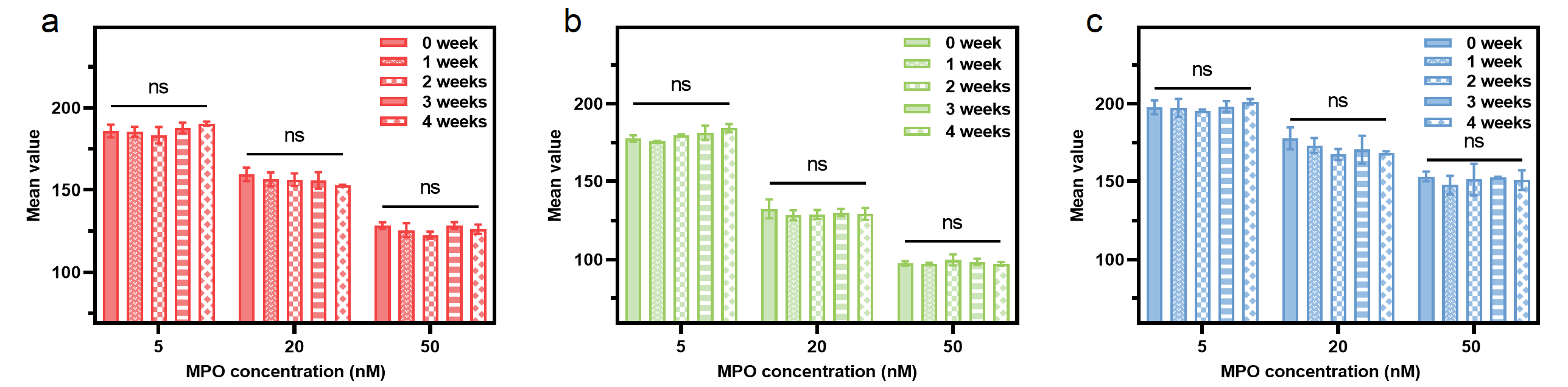


**Figure S9.** Red (a), green (b), and blue (c) mean values of the photograph of different concentration of MPO solution dripping on hydrogel sheet after different storage time (*n* = 3). Data are presented as mean ± s.d.. Statistical significance was determined by the one-way ANOVA. *P< 0.05, **P<0.01, ***P<0.001, ****P<0.0001.


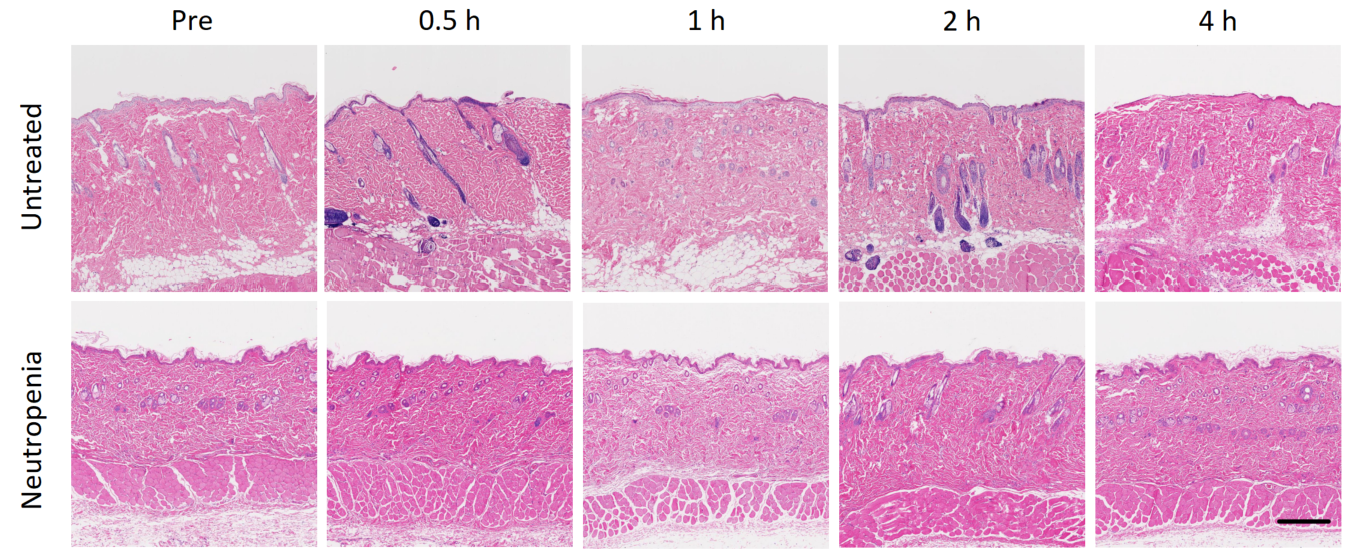


**Figure S10** Representative hematoxylin and eosin (H&E) stained sections of rat skin before and after pro-inflammation. Scale bar: 1 mm.


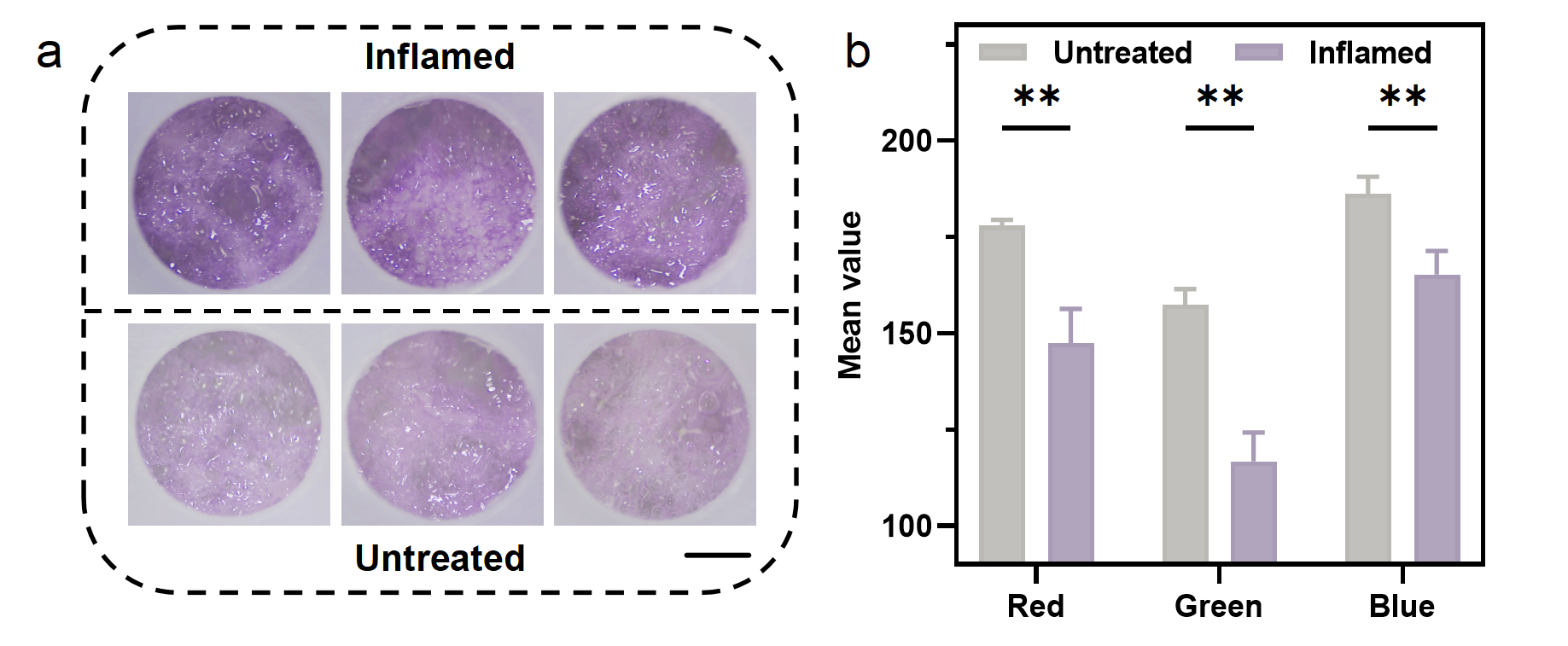


**Figure S11.** Detection ability of the microneedle patch in untreated and inflamed rats. (a) Photographs of the hydrogel sheet within the observation window after 2 hours of patch application with PMA and 5 minutes of ISF extraction on normal and inflamed rats (*n* = 3). Scale bar: 1 mm. (b) RGB mean values of the circular observation window in the picture in (a) (*n* = 3). In (b), data are presented as mean ± s.d.. Statistical significance was determined by the two-tailed Student’s t test. *P< 0.05, **P<0.01, ***P<0.001, ****P<0.0001.


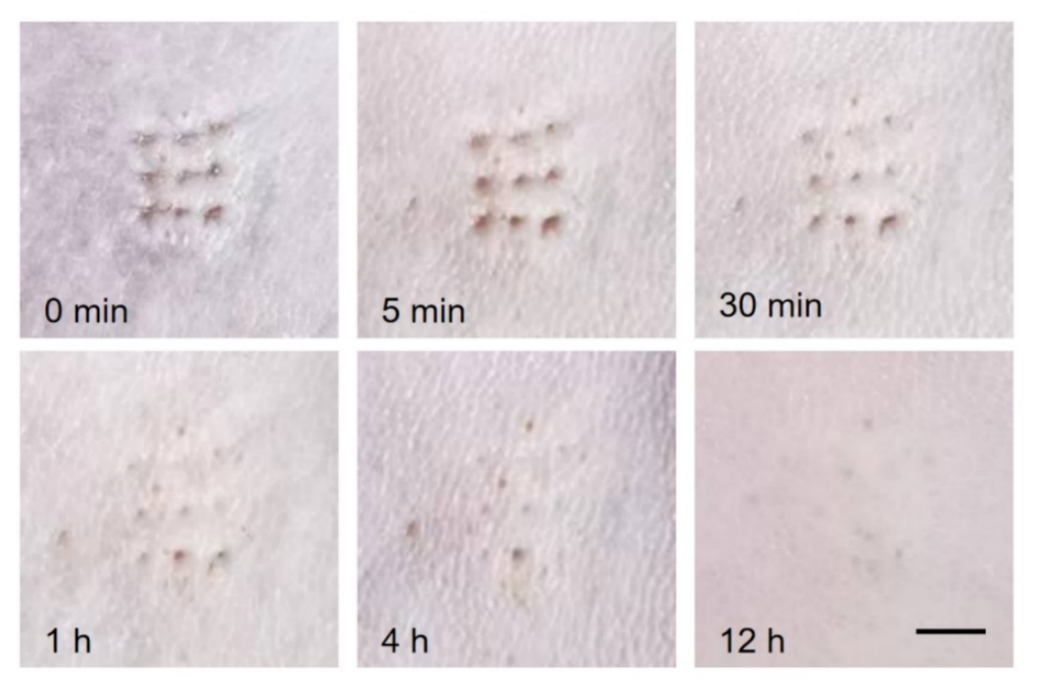


**Figure S12** Photographs of the hydrogel sheet within the observation window after 2 hours of patch application with PMA and 5 minutes of ISF extraction. Scale bar: 3 mm.


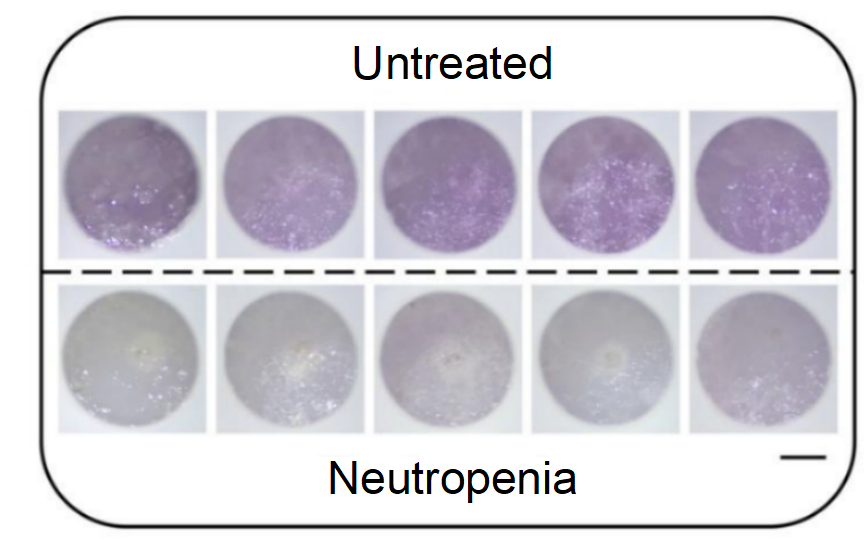


**Figure S13** Photographs of the hydrogel sheet within the observation window after 4 hours of patch application with histamine and 5 minutes of ISF extraction. Scale bar: 1 mm.


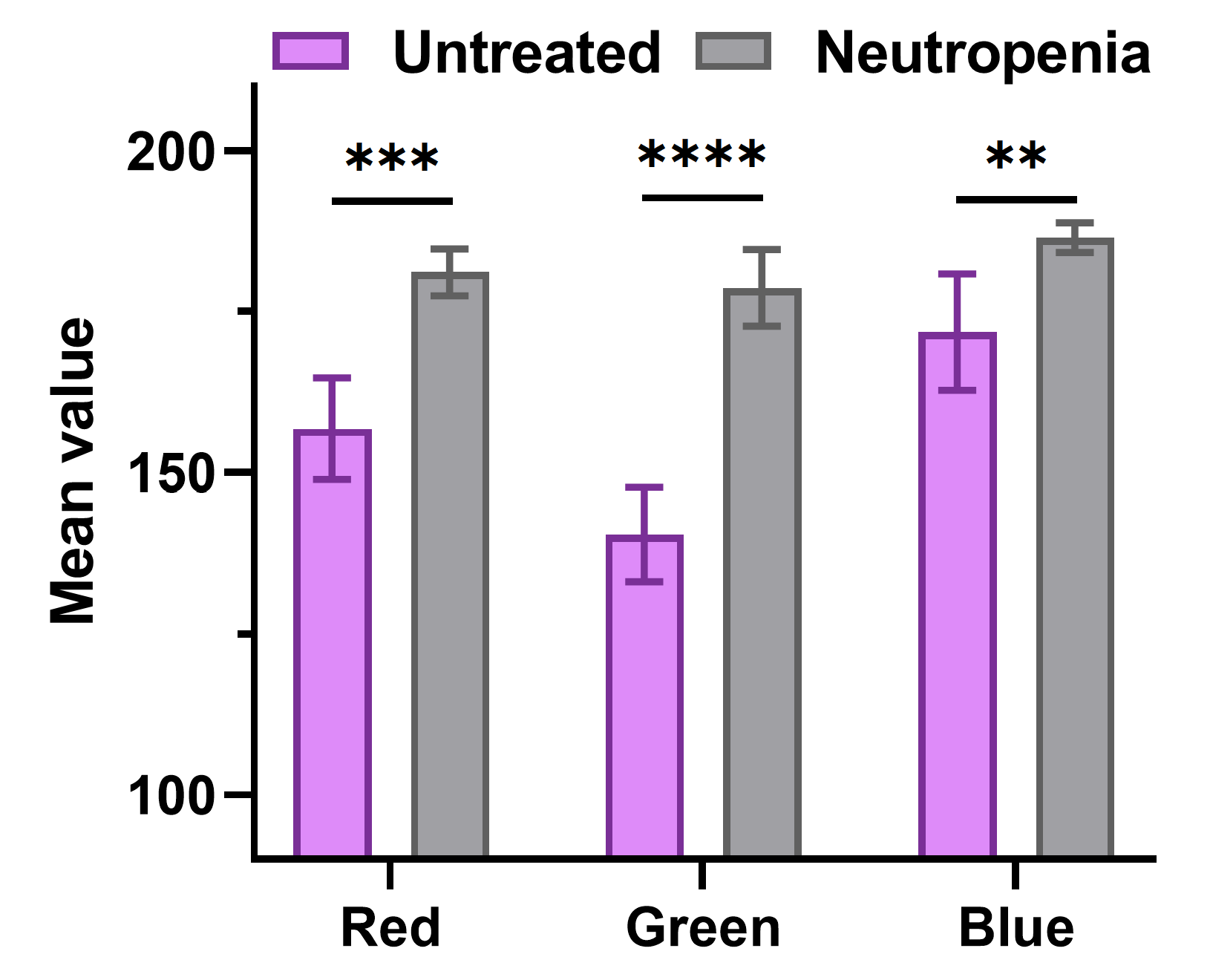


**Figure S14** RGB mean values of the circular observation window in the picture in Figure S13 (*n* = 5). Data are presented as mean ± s.d.. Statistical significance was determined by the two-tailed Student’s t test. *P< 0.05, **P<0.01, ***P<0.001, ****P<0.0001.


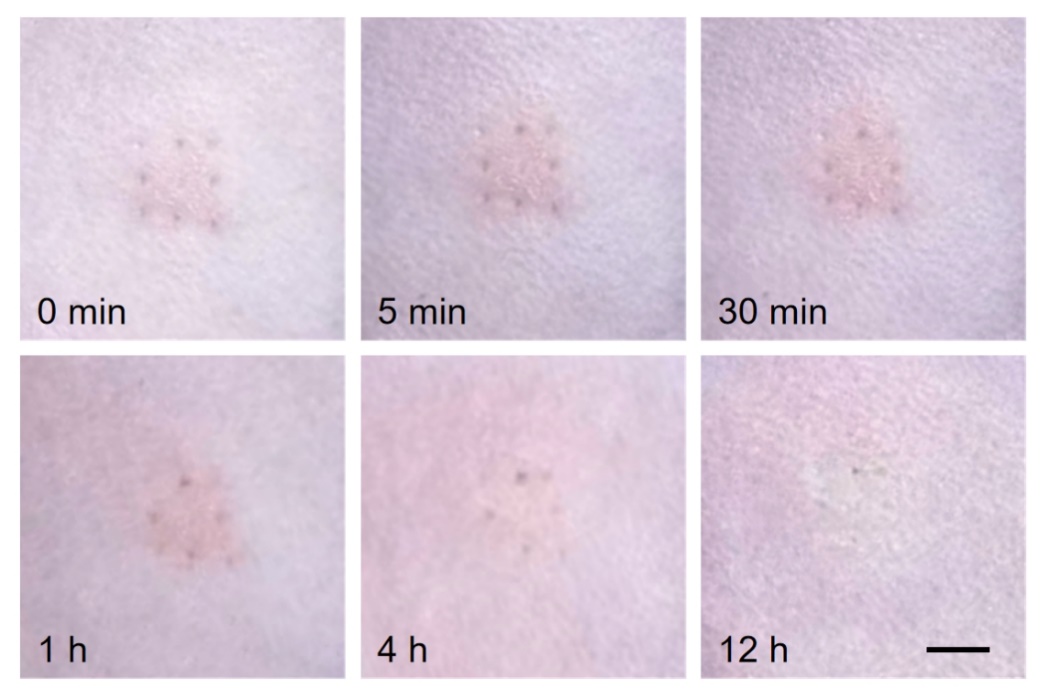


**Figure S15** Photographs of the hydrogel sheet within the observation window after 4 hours of patch application with histamine and 5 minutes of ISF extraction. Scale bar: 3 mm.


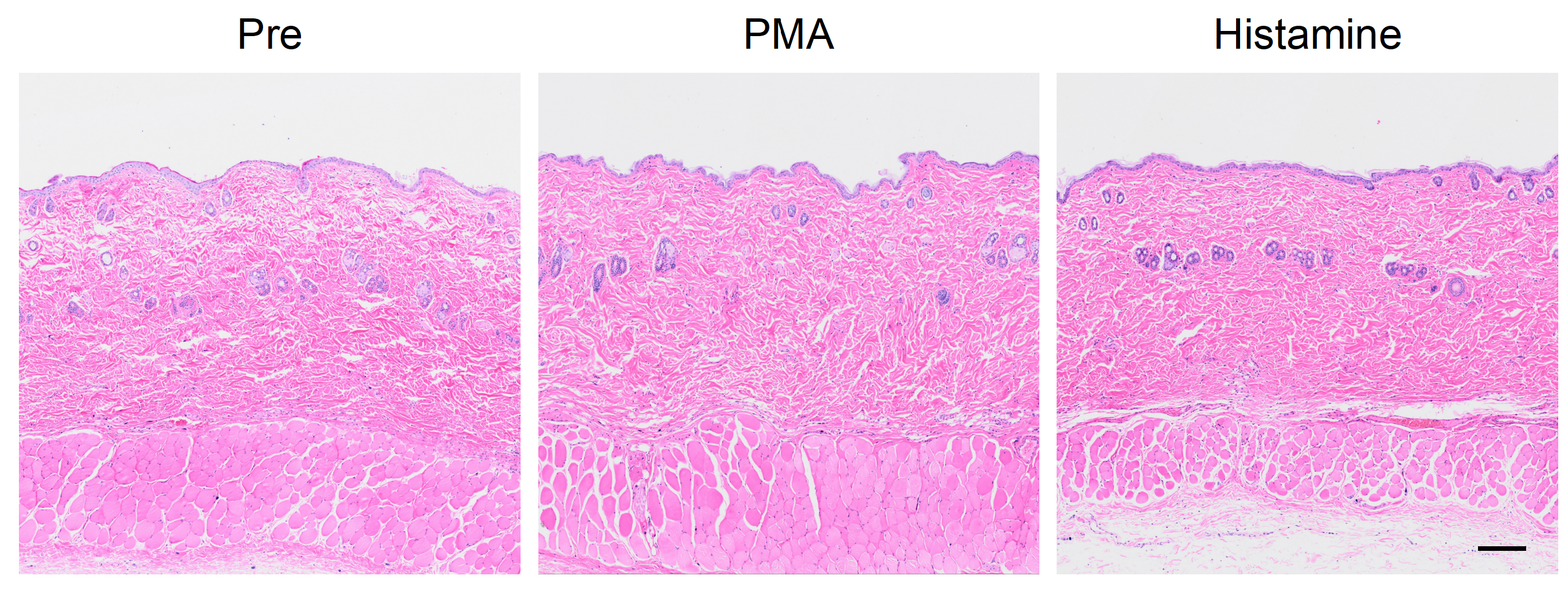


**Figure S16** Representative hematoxylin and eosin (H&E) stained sections of rat skin before and 24 h after application of microneedle patch loaded with PMA or histamine. Scale bar: 200 *μ*m.


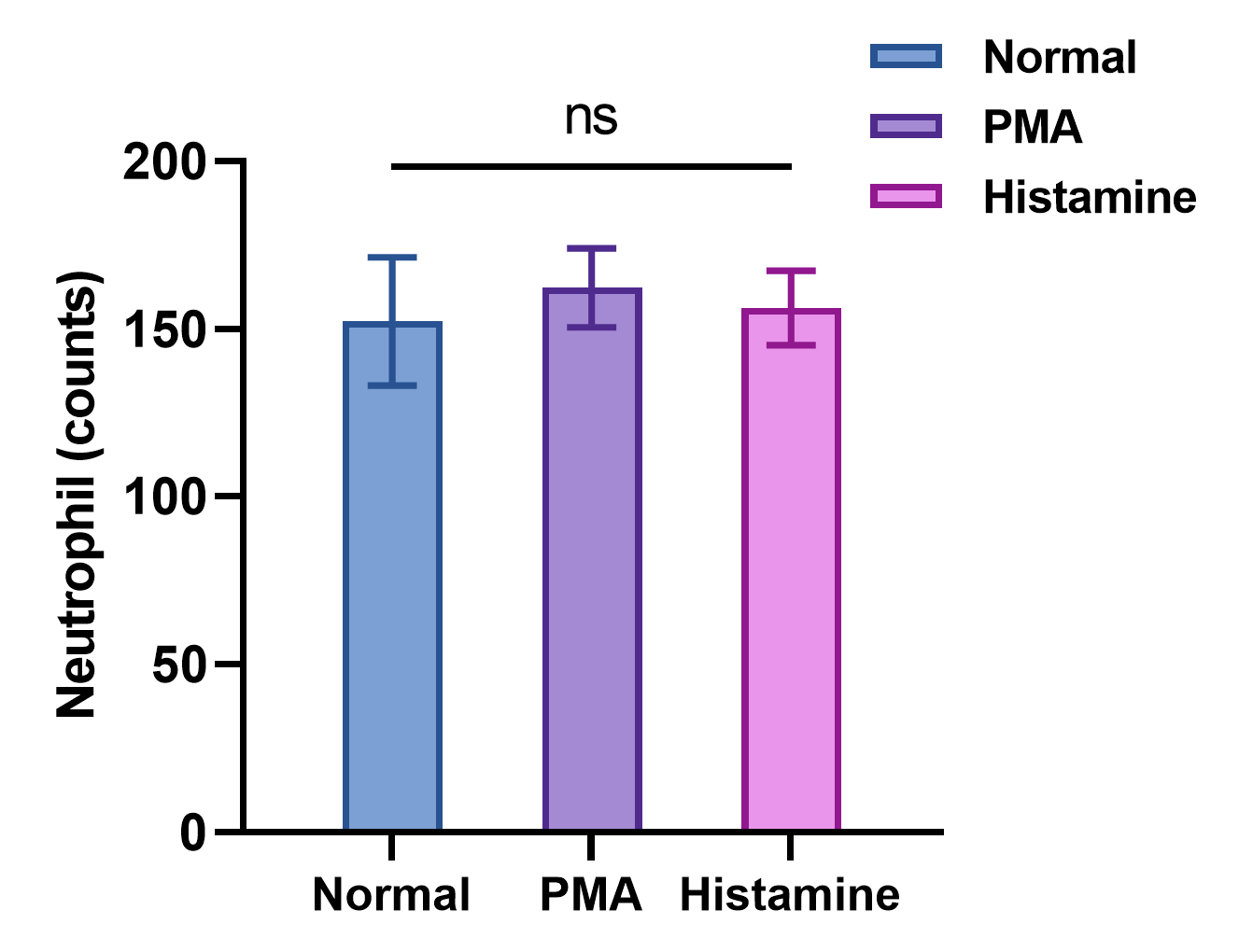


**Figure S17** Comparison of skin neutrophil level of rat skin before and 24 h after application of microneedle patch loaded with PMA or histamine (*n* = 3). Data are presented as mean ± s.d.. Statistical significance was determined by the one-way ANOVA. *P< 0.05, **P<0.01, ***P<0.001, ****P<0.0001.


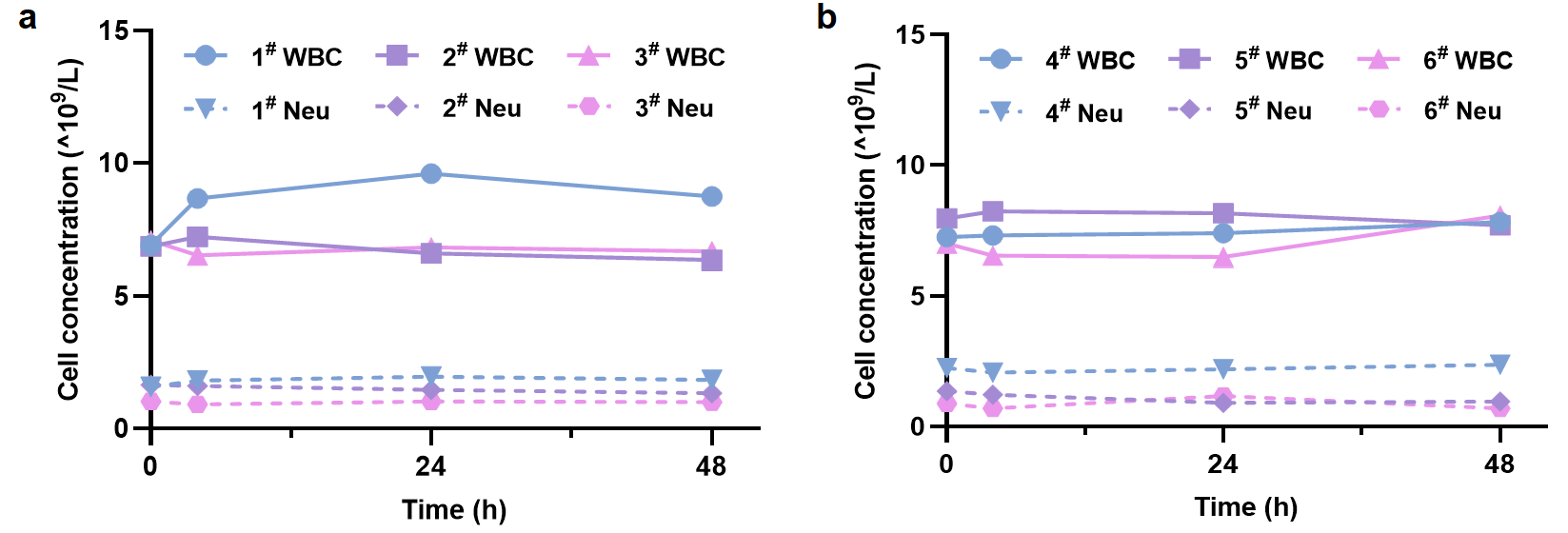


**Figure S18.** White blood cell count and neutrophil count before and after application of microneedle patch loaded with (a) PMA or (b) histamine (*n* = 3).

**Video S1** Video of PMA-loaded microneedle patch for detecting neutrophil levels in normal rats.

**Video S2** Video of PMA-loaded microneedle patch for detecting neutrophil levels in neutropenic rats.
